# Improving spinosad production by tuning expressions of the forosamine methyltransferase and the forosaminyl transferase to reduce undesired less active byproducts in the heterologous host *Streptomyces albus* J1074

**DOI:** 10.1186/s12934-023-02023-3

**Published:** 2023-01-19

**Authors:** Xiaochen Li, Ruofei Guo, Ji Luan, Jun Fu, Youming Zhang, Hailong Wang

**Affiliations:** State Key Laboratory of Microbial Technology, Institute of Microbial Technology, Helmholtz International Lab for Anti-infectives, Shandong University–Helmholtz Institute of Biotechnology, Shandong University, Binhai Rd 72, Qingdao, 266237 Shandong China

**Keywords:** Spinosad, Forosamine, *N,N*-dimethyltransferase, Forosaminyl transferase, Biosynthesis, Heterologous expression

## Abstract

**Background:**

Spinosad is a macrolide insecticide with the tetracyclic lactone backbone to which forosamine and tri-o-methylrhamnose are attached. Both the sugar moieties are essential for its insecticidal activity. In biosynthesis of spinosad, the amino group of forosamine is dimethylated by SpnS and then transferred onto the lactone backbone by SpnP. Because the spinosad native producer is difficult to genetically manipulate, we previously changed promoters, ribosome binding sites and start codons of 23 spinosad biosynthetic genes to construct an artificial gene cluster which resulted in a 328-fold yield improvement in the heterologous host *Streptomyces albus* J1074 compared with the native gene cluster. However, in fermentation of J1074 with the artificial gene cluster, the *N*-monodesmethyl spinosad with lower insecticidal activity was always produced with the same titer as spinosad.

**Results:**

By tuning expression of SpnS with an inducible promotor, we found that the undesired less active byproduct *N*-monodesmethyl spinosad was produced when SpnS was expressed at low level. Although *N*-monodesmethyl spinosad can be almost fully eliminated with high SpnS expression level, the titer of desired product spinosad was only increased by less than 38%. When the forosaminyl transferase SpnP was further overexpressed together with SpnS, the titer of spinosad was improved by 5.3 folds and the content of *N*-desmethyl derivatives was decreased by ~ 90%.

**Conclusion:**

*N-*monodesmethyl spinosad was produced due to unbalanced expression of *spnS* and upstream biosynthetic genes in the refactored artificial gene cluster. The accumulated *N*-desmethyl forosamine was transferred onto the lactone backbone by SpnP. This study suggested that balanced expression of biosynthetic genes should be considered in the refactoring strategy to avoid accumulation of undesired intermediates or analogues which may affect optimal production of desired compounds.

**Supplementary Information:**

The online version contains supplementary material available at 10.1186/s12934-023-02023-3.

## Background

**Fig. 1:**
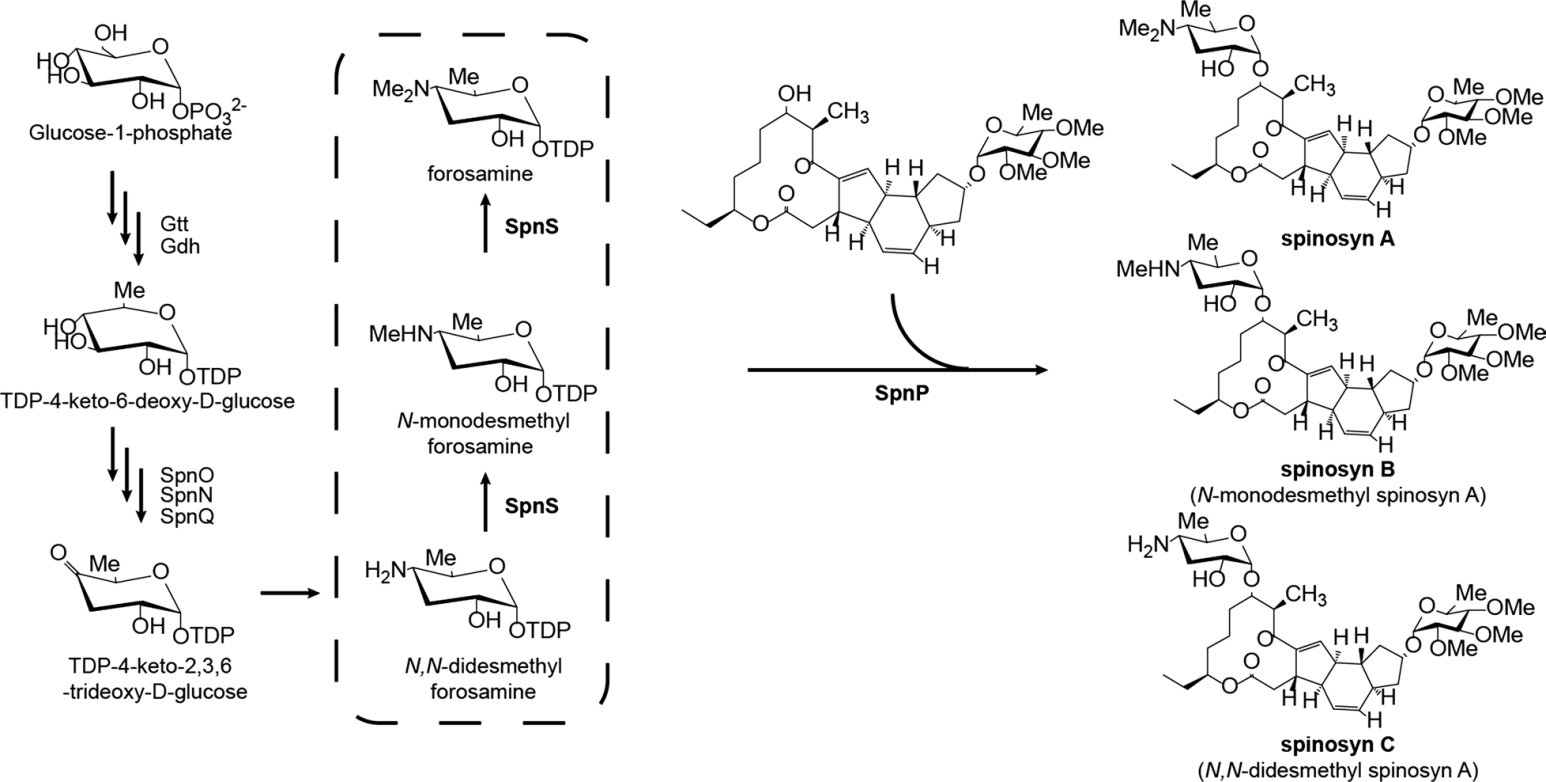
Biosynthesis of spinosyns **A**, **B** (*N*-monodesmethyl spinosyn A), and **C** (*N,N*-didesmethyl spinosyn A)

Spinosad, the mixture of spinosyns A and D, is a fermentation derived insecticide with high activity against a variety of chewing insect pests and exceptional safety profiles [1, 2]. During spinosad biosynthesis, a linear polyketide chain is made first by polyketide synthases SpnA-E [3], and then the tetracyclic aglycone is formed by cross-bridging enzymes SpnF, J, L, M [4]. Rhamnose is biosynthesized from glucose-1-phosphate by Gtt, Gdh, Epi, and Kre proteins, added to the aglycone by the transferase SpnG [5] and then o-methylated by SpnH, I, K [6] to form the pseudoaglycone. Forosamine biosynthesis shares the common first two steps with rhamnose [7] producing TDP-4-keto-6-deoxy-d-glucose which is further converted into TDP-d-forosamine by SpnO, N, Q, R, and S. *N*-methylation of the forosamine catalyzed by SpnS occurs in a stepwise manner and the monomethylated product (*N*-monodesmethyl forosamine) can be released from the active site of SpnS [8]. Forosamine is transferred onto the pseudoaglycone by SpnP [9] to finally form spinosad. SpnP can also transfer *N*-monodesmethyl and *N,N*-didesmethyl forosamines to the pseudoaglycone to produce *N*-monodesmethyl and *N,N*-didesmethyl spinosad [8] (Fig. 1). The insecticidal activities of *N*-monodesmethyl and *N,N*-didesmethyl spinosad are both lower than spinosad. The LC_50_ of spinosyn A to *Heliothis virescens* larvae are 0.3 mg L^− 1^, while the LC_50_ of *N*-monodesmethyl spinosyn A (spinosyn B) and *N,N*-didesmethyl spinosyn A (spinosyn C) are 0.4 mg L^− 1^ and 0.8 mg L^− 1^ respectively [10]. The LC_50_ of *N*-monodesmethyl spinosyn D to *Heliothis virescens* larvae is seven times higher than that of spinosyn D (5.6 vs. 0.8 mg L^− 1^) [10]. Therefore, *N*-desmethyl spinosad is undesired byproduct in the fermentation.

Because the spinosad native producer *Saccharopolyspora spinosa* is difficult to genetically manipulate, its gene cluster has been cloned and expressed in heterologous hosts such as *S. erythraea* [11], *S. coelicolor* [12], and *S. albus* [13, 14]. Previously, we constructed an artificial gene cluster in which the 23 spinosad biosynthetic genes were grouped into 7 operons under control of *Streptomyces* constitutive strong promoters [14]. When the artificial gene cluster was expressed in *S. albus* J1074, the yield of spinosad was improved 328 folds compared with the native gene cluster. Besides spinosad, the less active byproduct *N*-monodesmethyl spinosad was also detected at the same titer as spinosad in the fermentation broth of *S. albus* J1074 containing the artificial gene cluster.

Tuning expression of biosynthetic genes is an important way to reduce undesired compound production or enhance desired compound production. For example, in erythromycin A fermentation, erythromycins B and C are recognized as undesired byproducts because they are much less active and cause greater side effects [15]. Chen et al. nearly completely eliminated erythromycins B and C and improved the titer of erythromycin A by 25% by tuning expression of two tailoring enzymes, the P450 hydroxylase EryK and *S*-adenosylmethionine-dependent *O*-methyltransferase EryG [16]. In this study, we revealed that *N*-desmethyl spinosad was produced due to unbalanced expression of the forosamine methyltransferase gene *spnS* and upstream biosynthetic genes which caused accumulation of *N*-desmethyl forosamine. By tuning expression of *spnS*, we reduced the content of undesired less active byproduct *N*-desmethyl spinosad by more than 90% in the fermentation broth of *S. albus* J1074. Furthermore, the yield of desired product spinosad was increased by 5.3 folds to 5.8 mg L^− 1^ through overexpressing *spnS* together with the forosamyltransferase gene *spnP*.

## Results and discussion

### Expression of the forosamine methyltransferase gene *spnS* affected production of spinosad and its *N-*desmethyl derivatives

**Fig. 2:**
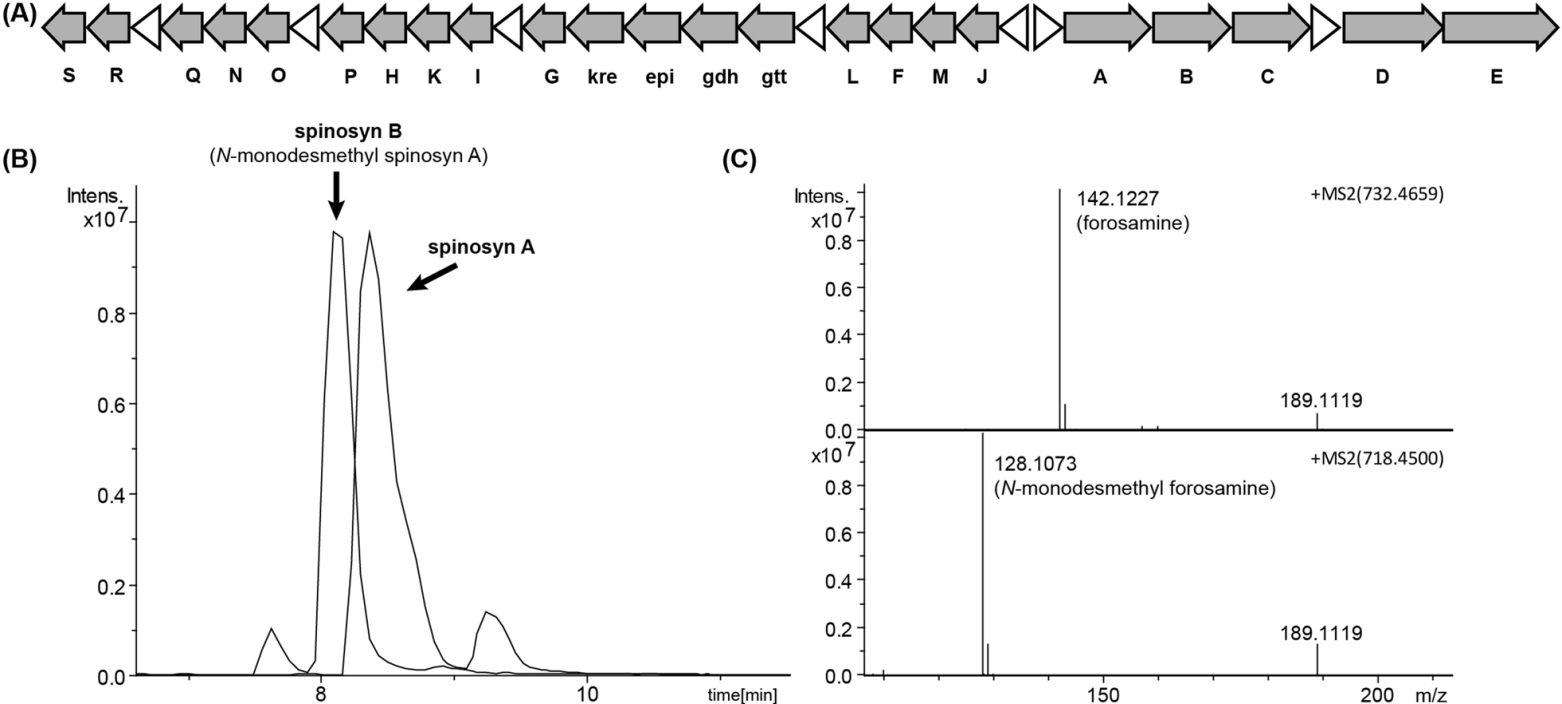
The spinosad 7-operon artificial gene cluster and its products. **A** Organization of the 7-operon artificial gene cluster. **B** HPLC-MS analysis (Base Peak Chromatogram) of spinosyns A and B produced in *S. albus* J1074 containing the 7-operon artificial gene cluster. **C** The MS^2^ fragmentation patterns of spinosyn A (top) and spinosyn B (down) produced in *S. albus* J1074. The fragment ion at *m/z* 142.1 and 128.1 are the characteristic forosamine and *N-*monodesmethyl forosamine fragments respectively. 189.1 is the characteristic trimethylrhamnose fragments [17]

Previously, we constructed an artificial gene cluster for efficient heterologous spinosad production in *S. albus* J1074 [14]. The artificial gene cluster consisted of all 23 spinosad biosynthetic genes grouped into 7 operons under control of strong constitutive promoters. In the fermentation broth of *S. albus* J1074 harboring the 7-operon artificial gene cluster (7op), besides the desired product spinosad, the less active analogue *N-*monodesmethyl spinosad was also detected (Fig. 2). The titer of *N-*monodesmethyl spinosyn A (spinosyn B) is the same as that of spinosyn A. In the forosamine biosynthesis, dimethylation of its amino group by SpnS occurs in a stepwise manner. The intermediates *N,N*-didesmethyl forosamine and *N*-monodesmethyl forosamine can also be transferred onto the macrolide backbone by SpnP. We speculated that the *N-*monodesmethyl spinosad was produced due to accumulation of *N*-monodesmethyl forosamine caused by insufficient expression of SpnS.

**Fig. 3:**
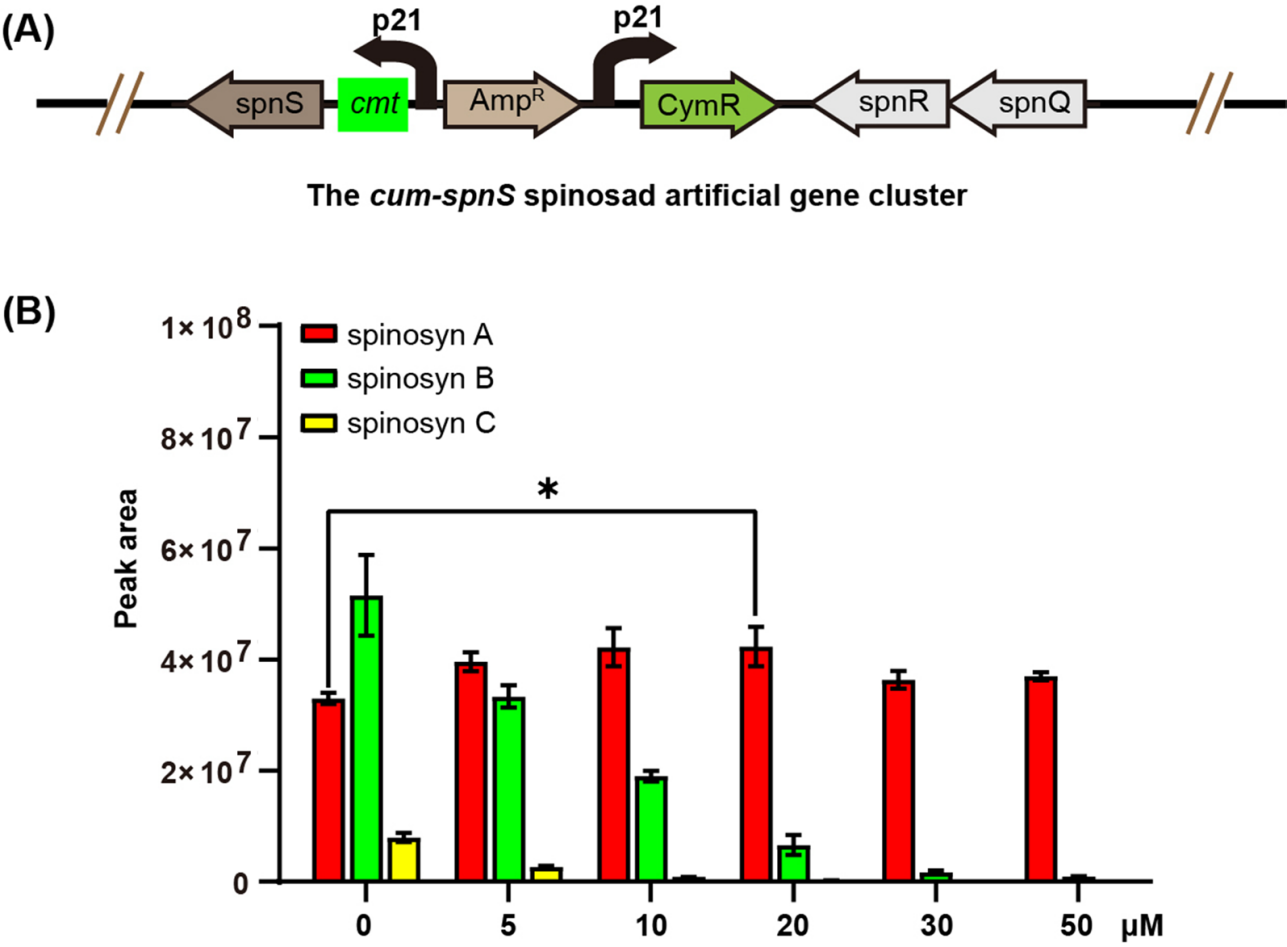
Effects of the *spnS* expression level on biosynthesis of spinosad and its *N-*desmethyl derivatives. **A** Schematic representation of the cymR-P21-cmt-spnS expression system. **B** Titers of spinosyns A, B and C under different concentration of cumate added in the fermentation medium. Each fermentation was done in triplicate (*n* = 3). Error bars represent standard deviation. Differences were analyzed by one-way ANOVA and *P* < 0.05 was considered statistically significant. ***P < 0.001, **P < 0.01, *P < 0.05

To determine the effects of *spnS* expression level on biosynthesis of spinosad and its *N-*desmethyl derivatives, we inserted the cumate-inducible cymR-P21-cmt expression system [18] upstream of the *spnS* gene in the artificial gene cluster by recombineering to generate the *cum-spnS* recombinant gene cluster (Fig. 3A). Recombineering is a DNA engineering technique which uses homologous recombination mediated by phage proteins in *E. coli* [19]. In the cymR-P21-cmt expression system, the CmyR repressor binding on the *cmt* operator placed downstream of the P21 promoter will block transcription of downstream genes. The inducer cumate added in the medium can bind CmyR and restore the transcription.

During fermentation of *S. albus* J1074 containing the *cum-spnS* gene cluster, when no cumate inducer was added into the medium, spinosyn A, *N*-monodesmethyl spinosyn A (spinosyn B), and *N,N*-didesmethyl spinosyn A (spinosyn C) were all produced. The titer of spinosyn B is higher than that of spinosyn A which is higher than that of spinosyn C (Fig. 3B and Additional file 1: Fig. S1). Because the cymR-P21-cmt promoter is not completely tight [20], low level leaky expression of SpnS caused production of *N*-monodesmethyl forosamine and *N,N*-didesmethyl forosamine which were both transferred onto the macrolide backbone by SpnP. As the cumate concentration in the fermentation broth increased, titers of spinosyns B and C decreased. When more than 20 µM of cumate was added into the fermentation medium, production of spinosyn C cannot be detected and production of spinosyn B decreased by more than 87% compared with the production without cumate addition. The lowest spinosyn B production, decreased by 98%, was obtained when 50 µM of cumate was added (Fig. 3B and Additional file 1: Fig. S1 and Table S1).

As the production of spinosyns B and C decreased, the production of spinosyn A increased when less than 20 µM of cumate was added. However, when more cumate was added, the yield of spinosyn A did not increase further as the production of spinosyns B and C decreased.

### Constitutive overexpression of *spnS* reduced production of *N*-desmethyl spinosyn A derivatives and improved spinosyn A production

**Fig. 4:**
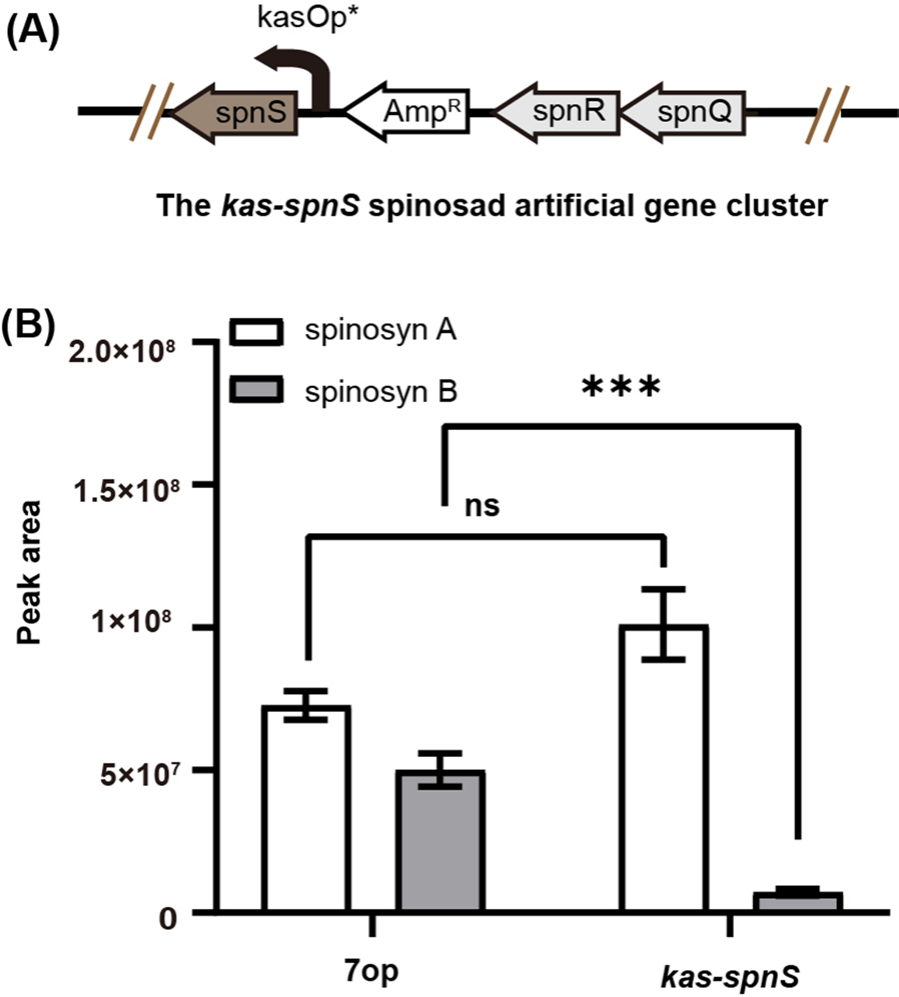
Effects of constitutive *spnS* overexpression on productions of spinosyns A and B. **A** Schematic representation of the *kasO*p*-*spnS* expression system. **B** Titers of spinosyns A and B from different gene clusters. Each fermentation was done in triplicate (*n* = 3). Error bars represent standard deviation. Differences were analyzed by one-way ANOVA and *P* < 0.05 was considered statistically significant. ***P < 0.001, **P < 0.01, *P < 0.05

In the cumate-inducible cymR-P21-cmt expression system, production of spinosyn A and its *N*-desmethyl derivatives was depended on the *spnS* expression level. We then replace the inducible cymR-P21-cmt expression system upstream of *spnS* with the strong constitutive *kasO*p* promoter [21] to generate the *kas-spnS* recombinant gene cluster (Fig. 4A). When the *kas-spnS* gene cluster was introduced into *S. albus* J1074, production of spinosyn A and its desmethyl derivatives are similar with the inducible cymR-P21-cmt expression system at high cumate concentration. Production of spinosyn C cannot be detected and production of spinosyn B decreased by 86% compared with the production from the original 7op gene cluster. The spinosyn A production from the *kas-spnS* gene cluster increased only 38% compared with the original 7op gene cluster (Fig. 4B).

### Constitutive overexpression of the forosamyltransferase gene *spnP* together with *spnS* further improved spinosyn A production

**Fig. 5:**
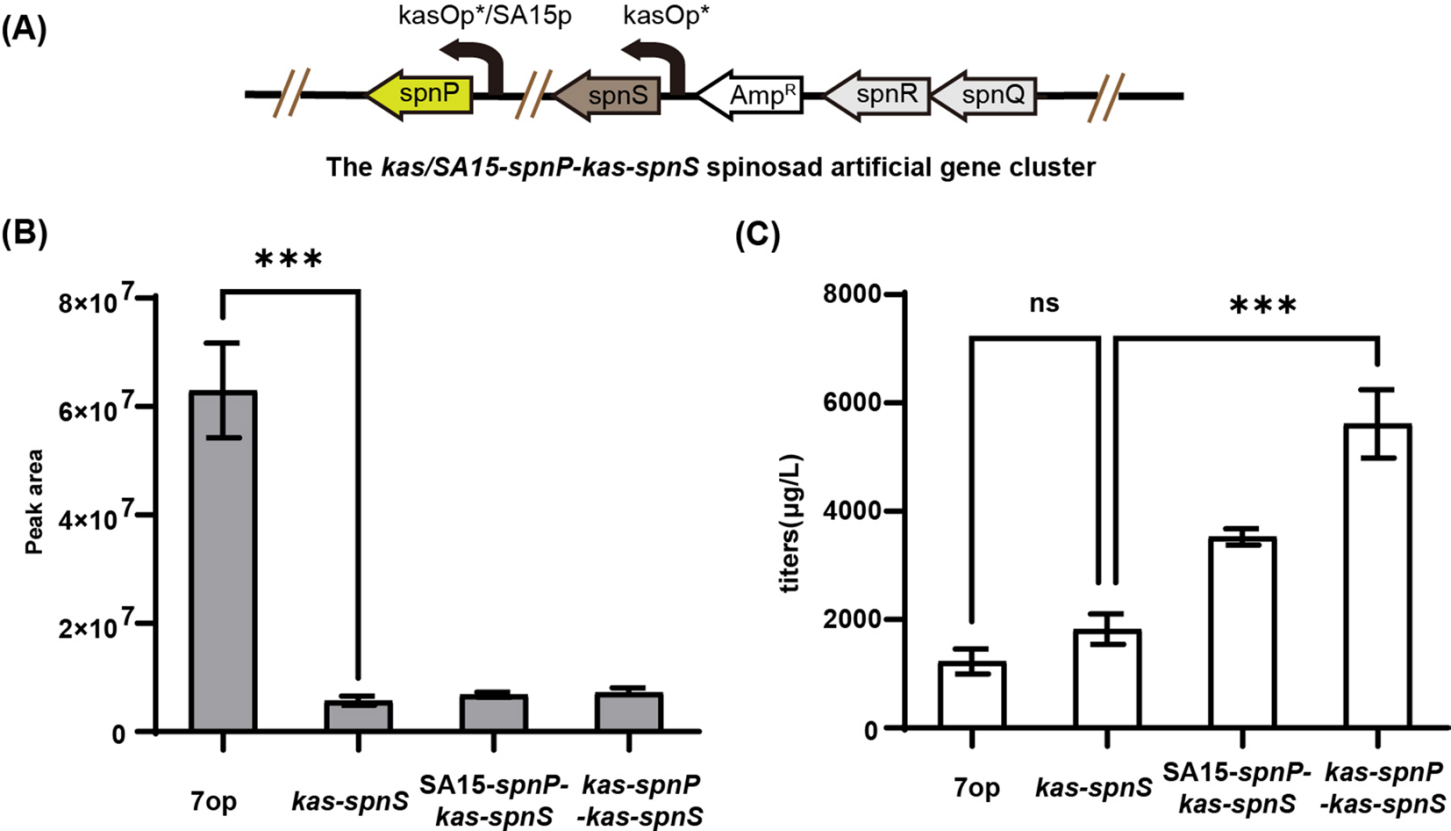
Effects of both *spnS* and *spnP* overexpression on spinosyn A and its *N*-desmethyl derivatives. **A** Schematic representation of the *kas-spnP-kas-spnS and SA15-spnP-kas-spnS* expression system. **B** Yield of spinosyn B from different gene clusters. **C** Yield of spinosad (spinosyns A and D) from different gene clusters. Each fermentation was done in triplicate (*n* = 3). Error bars represent standard deviation. Differences were analyzed by one-way ANOVA and *P* < 0.05 was considered statistically significant. ***P < 0.001, **P < 0.01, *P < 0.05

Above results suggested that although the amino group of forosamine was almost fully methylated in the *spnS* overexpression strain, a large quantity of fully methylated forosamine was not used for the biosynthesis of spinosad. We speculated that expression of the forosamyltransferase SpnP was not sufficient for fully transfer of forosamine to the spinosad pseudoaglycone. Two strong constitutive promoters (*kasO*p*, SA15p [22]) were inserted upstream of *spnP* in the *kas-spnS* gene cluster to generate recombinant gene clusters *kas-spnP-kas-spnS* and *SA15-spnP-kas-spnS* (Fig. 5A).

When the *kas-spnP-kas-spnS* and *SA15-spnP-kas-spnS* gene clusters were introduced into *S. albus* J1074 respectively, production of spinosyn C (*N,N*-didesmethyl spinosyn A) was not detected and production of spinosyn B (*N*-monodesmethyl spinosyn A) was low and similar with that in the *kas-spnS* strain (Fig. 5B). The titers of spinosyn A in the *kas-spnP-kas-spnS* and *SA15-spnP-kas-spnS* strains were 4.4 and 3.1 times higher than that in the *kas-spnS* strain, respectively. Finally, the spinosad (spinosyns A and D) production in *S. albus* J1074 was increased to 5.8 ± 0.4 mg L^− 1^ when both *spnS* and *spnP* were overexpressed under control of the *kasO*p* promoter (Fig. 5 C). This suggested that enhanced expression of SpnP significantly promoted transfer of forosamine to the pseudoaglycone and make the spinosad biosynthesis much more efficient.

## Conclusion

A polyketide assembly line often produces multiple structurally related compounds, such as avermectins [23], erythromycins [15] and spinosyns [2]. Biosynthesis of less active components will compete substrates and energy with the most active components, therefore, eliminating the production of byproducts is important for enhancing the titer of the desired compounds. In this study, we improved spinosad production by tuning expressions of the forosamine methyltransferase and the forosaminyl transferase to reduce undesired less active *N-*desmethyl byproducts in the heterologous host *S. albus* J1074. On the other hand, fine tuning expression of tailoring enzymes can channel the biosynthesis to specific analogues such as them with different methylation status which is helpful for diversification of structures.

Gene cluster reconstruction has been widely employed in optimizing production of natural products [24–26]. The less active *N-*desmethyl spinosad byproduct was accumulated due to unbalanced expression of the forosamine methyltransferase gene and upstream biosynthetic genes when we refactored the spinosad gene cluster. Therefore, balanced expression of biosynthetic genes should be considered in the reconstruction strategy to avoid accumulation of undesired intermediates or analogues.

## Methods

### Bacteria strains, and culture conditions

Bacteria strains and plasmids used in this study were listed in Additional file 1: Table S2. *Escherichia coli* strains were grown at 37 °C in Luria-Bertani (LB) medium. *Streptomyces albus* J1074 were cultured at 30 °C on mannitol soya flour agar plates for spore preparation and conjugation. Concentrations of antibiotics used in this study were: chloramphenicol, 15 µg mL^− 1^; kanamycin, 15 µg mL^− 1^; ampicillin, 100 µg mL^− 1^; apramycin, 20 µg mL^− 1^ spectinomycin,60 µg mL^− 1^ for *Escherichia coli* and 50 µg mL^− 1^ of apramycin ;100 µg mL^− 1^ of trimethoprim lactate salt for *Streptomyces*.

### DNA manipulation

The cmyR-amp^R^-P21-cmt cassette was synthesized on the pUC57 vector by GENEWIZ (Suzhou, China). The cmyR-amp^R^-P21-cmt cassette flanked with homology arms was amplified by PCR using cum-1 and cum-2 (Additional file 1: Table S3). Then the cmyR-amp^R^-P21-cmt cassette was inserted upstream of the *spnS* gene on pBAC-spnNEW [14] by recombineering [19]. Correct pBAC-spnNEW-cum-spnS recombinants were identified by the ApaLI restriction analysis (Additional file 1: Fig. S2).

The ampicillin resistance gene (*amp*^*R*^), the *kasO*p* promoter and homology arms were fused with overlap extension PCR using amp-1 & 2 and kas-1 & 2 respectively (Table S3). Then the amp^R^-*kasO*p* cassette was inserted upstream of the *spnS* gene on pBAC-spnNEW [14] by recombineering [19]. Correct pBAC-spnNEW-kas-spnS recombinants were identified by the XhoI restriction analysis (Additional file 1: Fig. S2).

The spect-ccdB cassette fanked with two PacI restriction sites and homology arms was amplified with PCR using spect-1 and spect-2 (Additional file 1: Table S3) and the pR6K-spect-ccdB plasmid as the template. The hygromycin resistance gene on pBAC-spnNEW-kas-spnS was replaced with the spect-ccdB cassette using recombineering in *E. coli* GBred-gyrA462. Correct pBAC-spnNEW-kas-spnS-spect-ccdB recombinants were identified by the MscI restriction analysis (Additional file 1: Fig. S2).

The *spnP* gene, the *kasO*p* promoter or the SA15p promoter and homology arms were fused with overlap extension PCR using spnP-1& 2, kas-1 & 3 and SA15-1 & 2 respectively (Additional file 1: Table S3). Then the *kasO*p*/SA15p-spnP PCR product and PacI digested pBACspn-kas-spnS-spect-ccdB were recombined using linear-linear homologous recombination [27]. Correct pBAC-spnNEW-kas-spnS-*kasO*p*/SA15p-spnP were identified by the BamHI restriction analysis (Additional file 1: Fig. S2).

Above constructed spinosad expression vectors were transformed into *S. albus* J1074 by conjugation [28] for fermentation and high performance liquid chromatography–mass spectrometry analysis.

### Fermentation and high performance liquid chromatography–mass spectrometry analysis of spinosad from engineered *Streptomyces* strains

*Streptomyces* strains were inoculated into 50 mL tryptic soy broth in 250-mL flasks as the seed culture and incubated at 30 °C with 220 rpm shaking for 3–4 days. 600 µL (1:50 dilution) seed culture was transferred into 30 mL fermentation broth (4% W/V glucose, 1% W/V glycerol, 3% W/V soluble starch, 1.5% W/V soytone, 1% W/V beef extract, W/V 0.65% peptone, 0.05% W/V yeast extract, 0.1% W/V MgSO_4_, 0.2% W/V NaCl, 0.24% W/V CaCO_3_) in 250 mL flasks, and incubated at 30 °C with 220 rpm shaking for 10 days. The 5mL fermentation cultures extracted with 3 × acetonitrile vortexed for 20 min, incubated for 30 min at room temperature. Then, the extract was evaporated and redissolved in 1 mL methanol. 10 µL extract was used for HPLC-MS analysis as described previously [14]. To quantify the amount of spinosad produced in *S. albus* J1074, standard spinosad was purchased from Sigma-Aldrich (cat. no. 33,706).

## Supplementary Information

**Additional file 1: Figure S1.** SHPLC-MS Analysis (Base Peak Chromatogram) of *Streptomyces albus* J1074 with pBAC-spnNEW-cum-spnS under different cumate concentrations. **Figure S2.** Restriction analysis of recombinant BACs in this work. **Table S1.** Production of spinosyns A, B and C in *Streptomyces albus* J1074 with pBAC-spnNEW-cum-spnS under different cumate concentrations; **Table S2.** Strains and plasmids used in this work. **Table S3.** Primers used in this work.

## Data Availability

All data for this study are included in this published article and its additional file.
