# Supplementary material for: Improving spinosad production by tuning expressions of the forosamine methyltransferase and the forosaminyl transferase to reduce undesired less active byproducts in the heterologous host Streptomyces albus J1074
Source: Microb Cell Fact. 2023 Jan 19;22:15. doi: 10.1186/s12934-023-02023-3 (PMC9854174; doi:10.1186/s12934-023-02023-3)
Supplement: Supplementary file 1 — Additional file 1: Figure S1. SHPLC-MS Analysis (Base Peak Chromatogram) of Streptomyces albus J1074 with pBAC-spnNEW-cum-spnS under different cumate concentrations. Figure S2. Restriction analysis of recombinant BACs in this work. Table S1. Production of spinosyns A, B and C in Streptomyces albus J1074 with pBAC-spnNEW-cum-spnS under different cumate concentrations; Table S2. Strains and plasmids used in this work. Table S3. Primers used in this work. [file 12934_2023_2023_MOESM1_ESM.pdf]

Additional file

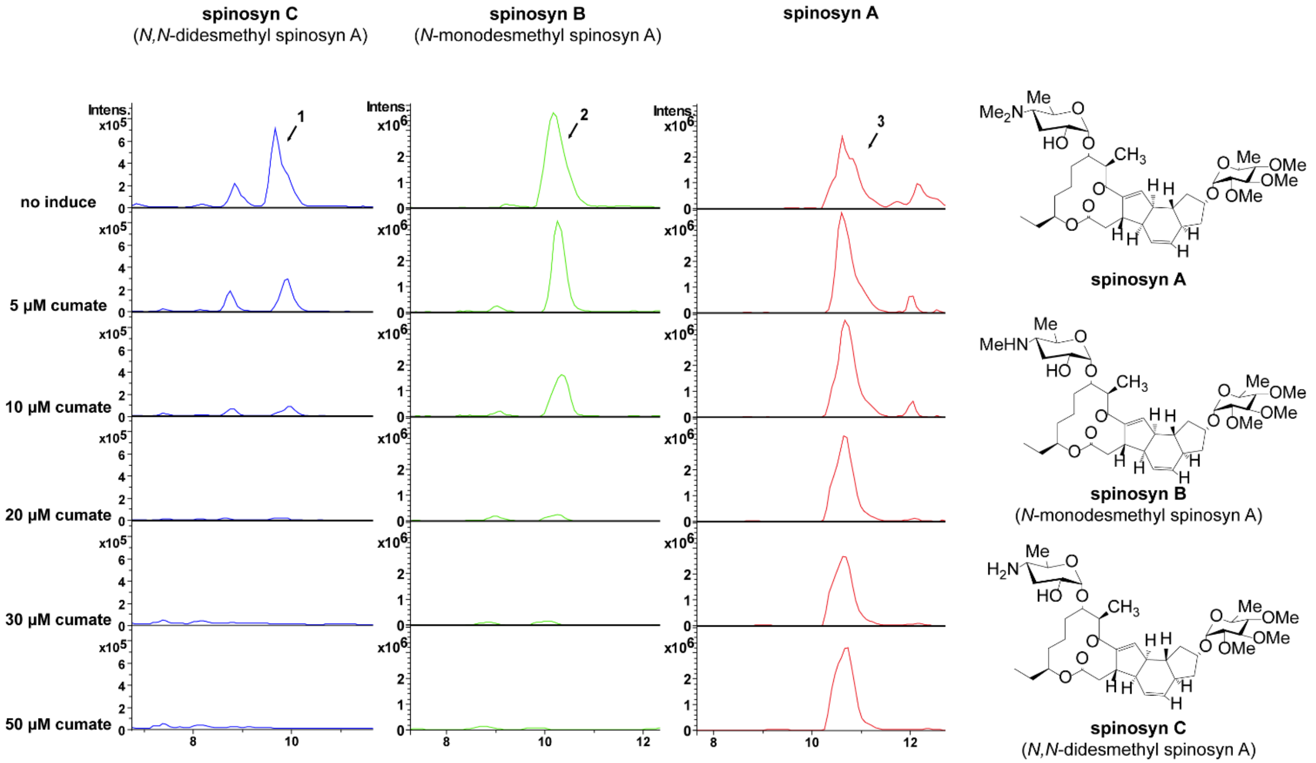

Fig. S1 HPLC-MS Analysis (Base Peak Chromatogram) of *Streptomyces albus* J1074 with pBAC-spnNEW-cum-spnS under different cumate concentrations.

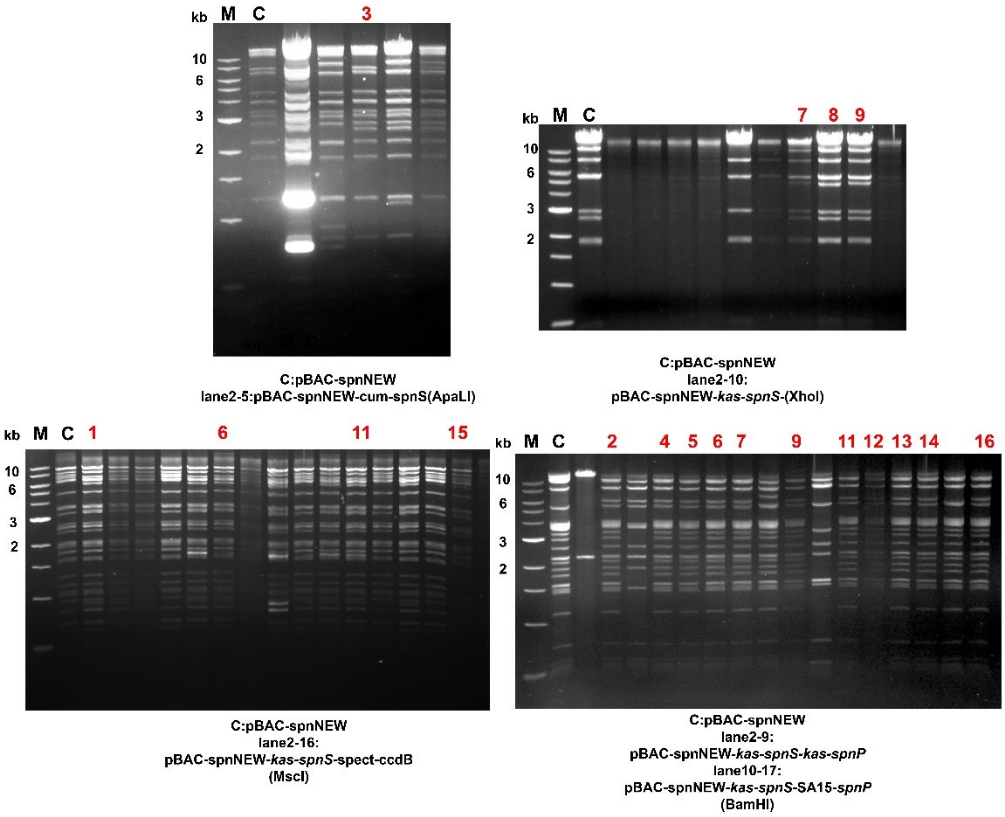

Fig. S2 Restriction analysis of recombinant BACs in this work. Correct clones are indicated with red number.

Table S1. Production of spinosyns A, B and C (peak area) in *Streptomyces albus* J1074 with pBAC-spnNEW-cum-spnS under different cumate concentrations.

	A	B	C
0	$3.3\pm0.1\times10^{7}$	$5.1\pm0.7\times10^{7}$	$7.9\pm0.2\times10^{6}$
5	$3.9\pm0.2\times10^{7}$	$3.3\pm0.1\times10^{7}$	$2.6\pm0.2\times10^{6}$
10	$4.2\pm0.2\times10^{7}$	$1.8\pm0.2\times10^{7}$	$8.4\pm0.2\times10^{5}$
20	$4.2\pm0.3\times10^{7}$	$6.5\pm0.8\times10^{6}$	$2.5\pm0.2\times10^{5}$
30	$3.6\pm0.2\times10^{7}$	$1.6\pm0.2\times10^{6}$	-
50	$3.7\pm0.3\times10^{7}$	$9.3\pm0.2\times10^{5}$	-

Table S2. Strains and plasmids used in this work.

Strains/Plasmids	Description	Sourse/Reference
**Strains**		
Escherichia coli GB05-red	DH10B, fhuA::IS2, ΔybcC, ΔrecET, P_{BAD}-γβαA	
Escherichia coli GBred-gyrA462	DH10B, fhuA::IS2, ΔybcC, ΔrecET, P_{BAD}-γβαA, gyrAArg462Cys	[1]
Escherichia coli GB05-dir	DH10B, fhuA::IS2, ΔybcC, ΔrecET, P_{BAD}-ETγA	
Streptomyces albus J1074		[2]
**Plasmids**		
pR6K-spect-ccdB	PCR template of spect-ccdB	This work
p15-cm-*kasO*p*-neongreen	PCR template of *kasO*p*	This work
pBAC-spnNEW	Containing refactored spinosad gene cluster .	[3]
pBAC-spnNEW-*cum-spnS*	Containing refactored spinosad gene cluster and *cum-spnS* cassete .	This work
pBAC-spnNEW-*kas-spnS*	Containing refactored spinosad gene cluster and *kas-spnS* cassete.	This work
pBAC-spnNEW-*kas-spnS-spect-ccdB*	Containing refactored spinosad gene cluster, *kas-spnS* and spect-ccdB cassete	This work
pBAC-spnNEW-*kas-spnS-kas-spnP*	Containing refactored spinosad gene cluster, *kas*-spnS-*kas-spnP* cassete	This work
pBAC-spnNEW-*kas-spnS*-SA15-*spnP*	Containing refactored spinosad gene cluster, *kas-spnS*-SA15-*spnP* cassete	This work

Table S3. Primers used in this work.

Name	Sequence (5'-3')	PCR templates
**Primers used in the plasmid construction of pBAC-spnNEW-cum-spnS.**		
cum-1	CCGAGGAGGTTTCTGCGAATGTGTCGCTCACGCGCGACATGTCCGTACCTCCGTTGCTCGACTAG	cumate-amp cassete
cum-2	AGCCAAAGATCACCACTCCGAAAGTGAGGGAGGAGGAACCCTCACCGCTTGAACTTGGCGTACCG	
**Primers used in the plasmid construction of pBAC-spnNEW-*kas*-spnS**		
kas-1	GGGCTGTATACCGAGGAGGTTTCTGCGAATGTGTCGCTCACGCGCGACATTAACTCCCCCAGTCCTGCACGCTGT	p15-cm-*kas*Op*-neongreen
kas-2	ATTTAGAAAAATAAACAAAGGATCCGTGTTCACATTCGAACCGTCTCTGC	
amp-1	TCAAAGCAGAGACGGTTCGAATGTGAACACGGATCCTTTGTTTATTTTTCTAAAT	pBR322-amp-ccdB-rpsL
amp-2	TTTAAATGGTACCTATATGAGTAAACTTGGTCTGACAGTTACCAATGCTTAATCAGTGAGGCAC	
**Primers used in the plasmid construction of pBAC-spnNEW-*kas*-spnS-spect-ccdB**		
spect-1	CCTTCGACGTGGTGAGCAAGGTGGACCACCAAGAGGTGGACAACGCGCTG *TTAATTAA* CAACTATGGATATAAAATAGGTA	pR6K-spect-ccdB
spect-2	TCCTGTGCGACGGTTACGCCGCTCCATGAGCTTATCGCGAATAAATACCT*TTAATTA* ATTTGTTCAAAAAAAAGCCCGCTC	
**Primers used in the plasmid construction of pBAC-spnNEW-*kas*-spnS- *kas*-spnP**		
kas-1	GGGCTGTATACCGAGGAGGTTTCTGCGAATGTGTCGCTCACGCGCGACATTAACTCCCCCAGTCCTGCACGCTGT	p15-cm-*kas*Op*-neongreen
kas-3	TGTTCACATTCGAACCGTCTCTGC	
spnP-1	CCAGGAGAATACGACAGCGTGCAGGACTGGGGGAGTTAATGATTCTTGGCATGCTTCCC	*S.albus* J1074 genome
spnP-2	AACTCCTGTGCGACGGTTACGCCGCTCCATGAGCTTATCGCGAATAAATACCTTCACGGATGGCCATCAGACTG	
**Primers used in the plasmid construction of pBAC-spnNEW- *kas*-spnS-SA15-spnP**		
S15p-1	CCTTCGACGTGGTGAGCAAGGTGGACCACCAAGAGGTGGACAACGCGCTGTCCGCGCCGCCGGCCCGACGGTGC	*S.albus* J1074 genome
S15p-2	GCGTAACCGTCGCACAGGA	
Spnp-3	CTCTCTTGCAGTGAACAAGTGGACTCATGAGGAGGAACCCATGATTCTTGGCATGCTTCCC	*S.albus* J1074 genome
Spnp-2	AACTCCTGTGCGACGGTTACGCCGCTCCATGAGCTTATCGCGAATAAATACCTTCACGGATGGCCATCAGACTG	

Underlined uppercase letters are the homologous arms of the recombineering. Italic letters are restriction site

REFERENCES

1. Wang H, Li Z, Jia R, Hou Y, Yin J, Bian X, et al. RecET direct cloning and Redab recombineering of biosynthetic gene clusters, large operons or single genes for heterologous expression. Nat Protoc. 2016;11(7):1175-90.

2. Chater KF, Wilde LC. Restriction of a bacteriophage of Streptomyces albus G involving endonuclease SalI. J Bacteriol. 1976;128(2):644-50.

3. Song C, Luan J, Cui Q, Duan Q, Li Z, Gao Y, et al. Enhanced heterologous spinosad production from a 79-kb synthetic multi-operon assembly. ACS Synth Biol. 2019;8(1):137-47.
